# Pulmonary nontuberculous mycobacterial disease in Florida and association with large-scale natural disasters

**DOI:** 10.1186/s12889-021-12115-7

**Published:** 2021-11-10

**Authors:** Shweta Kambali, Elena Quinonez, Arash Sharifi, Abdolrazagh Hashemi Shahraki, Naresh Kumar, Jayaweera Dushyantha, Mehdi Mirsaeidi

**Affiliations:** 1 Division of Pulmonary and Critical Care, University of Miami, Miami, Florida USA; 2 School of Medicine, University of Miami, Miami, Florida USA; 3 Rosenstiel School of Marine and Atmospheric Science, University of Miami, Miami, Florida USA; 4 Division of Pulmonary, Critical Care and Sleep, College of Medicine-Jacksonville, University of Florida, 653-1 8th St West, Jacksonville, Florida 32209 USA; 5 Department of Public Health Sciences, University of Miami, Miami, Florida USA; 6 Division of Infectious disease, University of Miami, Miami, Florida USA

**Keywords:** NTM, Nontuberculous, Mycobacteria, Florida, Hurricane

## Abstract

**Background:**

Little is known about the impact of the ecosystem disruption and its contribution on the non-tuberculosis mycobacteria (NTM) diseases (cases) rate in Florida (FL), a state with a high prevalence of NTM in the United States. We aimed to evaluate the epidemiological distribution of NTM in FL and identify its association with extreme weather events.

**Methods:**

We used OneFlorida Clinical Research Consortium dataset and extracted data on NTM cases using ICD codes 9- CM 031.0 and ICD-10 A31 during 2012–2018. The number of hurricanes during the study period which affected FL were extracted data from the National Hurricane Center (NHC) and the National Oceanic and Atmospheric Administration (NOAA).

**Results:**

Prevalence of NTM gradually increased during the study period. The rate was 2012: 14.3/100,000, 2015; 20.1/100,000 and 2018; 22.6/100,00 except in 2014 where there was an 8% decrease. The incidences were 2012; 6.5/100,00, 2015; 4.9/100,000 and in 2015; 5.4/100,000. Geographical analysis demonstrated a gradual expansion of the NTM cases in Alachua, and Marion Counties throughout the study period. Notably, the 2018 heat map showed higher prevalence of NTM in the northwestern, panhandle region of FL which had been absent in the heat maps for years 2012–2018. High number of the hurricanes was associated with the higher number of the new cases of NTM infection for years 2012, 2016–2018, while the lower number of the hurricanes was associated with the lower number of the new cases of NTM infection for years 2014–2015.

**Conclusion:**

The current study found the prevalence rates of NTM disease in FL rose from 2012 to 2018. A higher prevalence was seen following the hurricanes.

**Supplementary Information:**

The online version contains supplementary material available at 10.1186/s12889-021-12115-7.

## Background

Nontuberculous mycobacteria (NTM) are gram-positive, environmental bacteria that cause opportunistic infections in humans [1]. Mycobacteria are commonly found in water or moist soil environments. Pre-existing lung diseases (bronchiectasis, chronic obstructive pulmonary disease (COPD), and asthma), immunocompromised conditions, and certain genetic disorders [2–4], increase human susceptibility to NTM related disease.

In recent years, the incidence of pulmonary NTM disease has gradually increased in the United States (U.S.) [5] up to 8% each year [6, 7]. The prevalence of pulmonary NTM disease varies regionally, with higher rates in coastal areas [4]. The cause of this rise in some of the states can be attributed to favorable environmental conditions which promotes the survival of mycobacterium [8]. We recently reported higher period prevalence rates of pulmonary NTM in Puerto Rico, Florida (FL), and the District of Columbia [4] . Another statewide study also demonstrated a higher incidence and prevalence in FL as compared to other states, which possibly makes FL an NTM endemic area [6]. However, none of the studies have reported the correlation of change in trend of NTM cases with natural disasters.

Climate change is affecting environmental conditions, causing an increase in the risks of respiratory infections [9]. Similarly, it has also contributed to increase in NTM related disease caused by *M. abscessus*, *M. avium*, and *M. intracellulare* [10]. Some of the factors include soil pH, low to moderate (− 14 °C to 38 °C, with no light) temperature, and rainfall which are associated with the survival of mycobacteria in the environment [10, 11].

Increasing severity of storms in recent decades has been linked to climate change [12]. Tropical storms and hurricanes cause environmental changes, including flooding, increased standing waters, and increased soil moisture and are known to impact human health [13–15]. Such changes have been shown to increase lung diseases, including pneumonia with usual and unusual pathogens including NTM [16]. Individuals’ time of exposure during a natural disaster may be a central determinant to their acquisition of pulmonary NTM.

Although change in soil temperature and precipitation (as an indicator for soil moisture) are associated with the survival of NTM organisms [17], there is little data on the trends of pulmonary NTM during or after major hurricanes in FL. The purpose of the present study was to determine the current epidemiology of pulmonary NTM in FL in relation to the occurrence of hurricanes by evaluating incidence, prevalence, and geographic distribution of NTM annually from 2012 to 2018.

## Methods

**Fig. 1:**
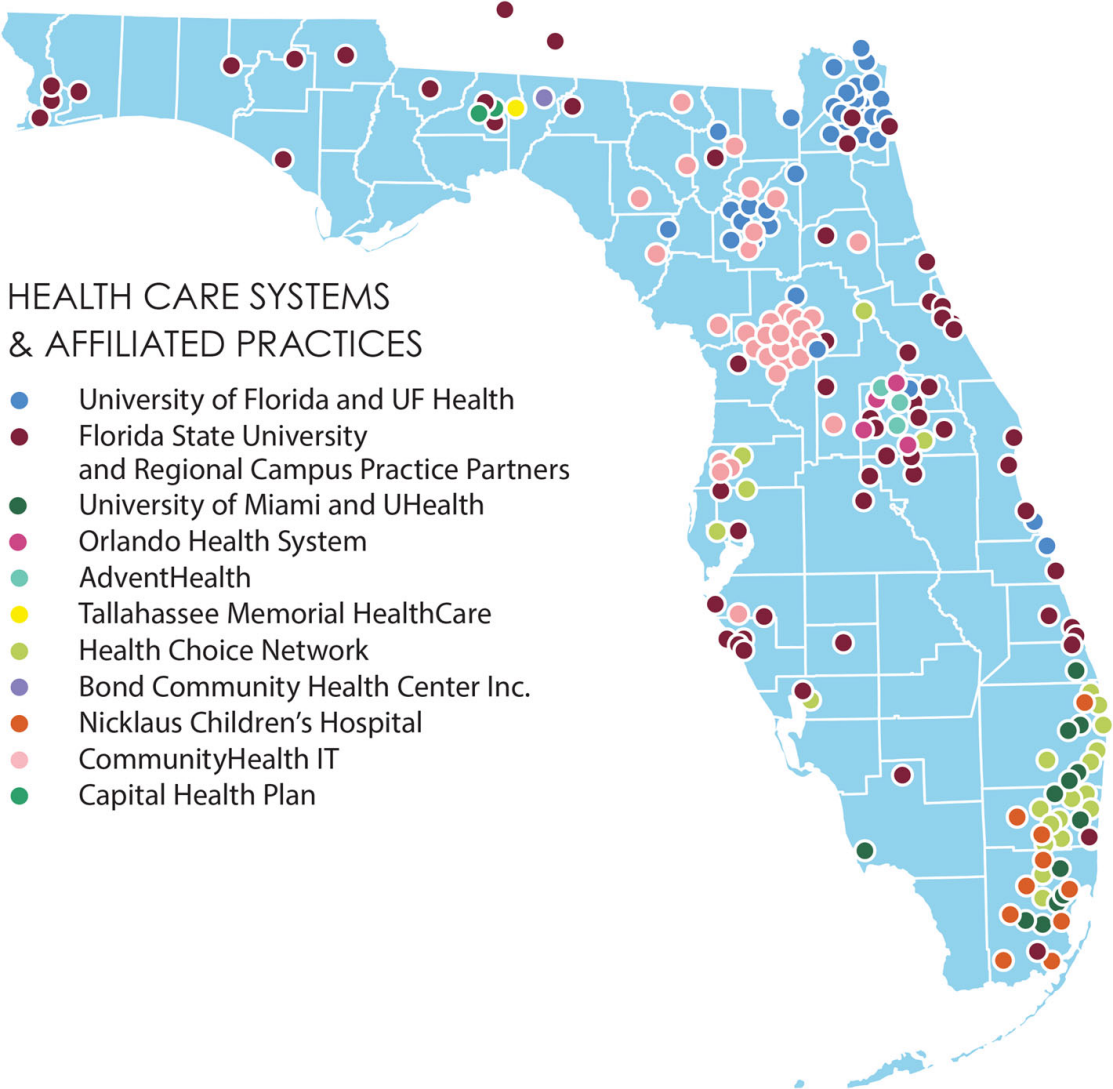
Shows OneFlorida Clinical Research Consortium in Florida. The image was generated by the authors using Microsoft Editor (Office 365) by adding colored dots representing 13 clinical data research networks across Florida

The area of Florida is 65,757.70 sq. miles (170,312 sq. km). According to U.S. 2020 Census results, Florida’s population in 2020 was around 21.5 million and is now estimated to be 21.6 million in 2021. This state is the third most populous state in the U.S. with the population density of 397.2 residents per square mile. This Clinical Research Consortium is a statewide research network that includes patients and caregivers, clinicians, researchers, state agencies, human-subject protection experts, and stakeholders in research initiatives across FL. OneFlorida cover ~ 15.3 million patients (> 40% of Floridians) in 67 counties, 1240 clinical practices, 4100 physician providers, and 22 hospitals (Fig. 1).

We requested NTM data from the OneFlorida Clinical Research Consortium dataset. NTM disease was diagnosed according to the following criteria suggested by Griffith et al., [18] and the data for each person was recorded in OneFlorida: (1) chest radiograph or, in the absence of cavitation, chest high-resolution computed tomography (HRCT) scan; (2) three or more sputum specimens for acid-fast bacilli (AFB) analysis; and (3) exclusion of other disorders, such as tuberculosis (TB)”. Since the consortium was only established in 2012, the study timeline ranged between 2012 to end of 2018. The study sample was extracted from the database using ICD codes 9- CM 031.0 and ICD-10 A31 for pulmonary NTM (infection due to other mycobacteria) as primary diagnosis. ICD codes were used to identify common comorbid conditions, including bronchiectasis, COPD, diabetes, cystic fibrosis (CF), HIV, lung transplant recipients (ICD codes are in supplementary materials) as secondary diagnosis. CPT codes were used for procedures, such as lung transplants; Logical Observation Identifiers Names and Codes (LOINC) were used to extract laboratory results; RxNorm (provides normalized names for clinical drugs) and concept unique identifiers (RXCUIs) were used to collect laboratory and medication data (Find ICD codes in supplementary materials). Further, demographics including the age, sex, race, and zip code of residence were extracted to identify the geographic distribution of the NTM disease. The number of hurricanes during the study period which affected Florida were extracted from National Hurricane Center (NHC) and from the National Oceanic and Atmospheric Administration (NOAA).

The 30-year average (1981–2010) surface temperature and precipitation variation over FL were made using the climate data from the PRISM climate group, Oregon State University (https://prism.oregonstate.edu/normals/). The surface temperature and precipitation maps were overlaid by the distribution of the total number of pulmonary NTM cases in FL. A total number of pulmonary NTM cases was calculated by the summation of all cases reported for any given ZIP code from 2012 to 2018. The NTM distribution maps for FL were generated by utilizing the XY coordinates of zip codes and the NTM prevalence for each year (from 2012 to 2018). The data grids for the zip code based pulmonary NTM prevalence distribution maps were generated using the Kriging method, and the grids were left blank outside the hull of data. Filled contour maps were generated using the medium smoothing method.

Time series data from the monthly number of existing pulmonary NTM diagnoses from January 2012 through December 2018 was plotted. A polynomial regression was used to find overall trends in the time series and the periodic variation. Time series data for the annual number of existing pulmonary NTM cases in Miami (the most populous city in South Florida) was made by combining the data from all zip codes in each area for each year. FL population distribution of age was used to calculate age-adjusted incidence and prevalence in 100,000 residents for each index year. To determine the effects of pulmonary NTM in COPD admission in FL, the Florida Environmental Public Health Tracking Program was used to generate a map for the age-adjusted rate of COPD Hospitalizations per 10,000 (2018) [19]. The graphs were generated using GraphPad Prism version 9.0.0. Heat maps were generated using Kriging method in R environment (version 4.1.2).

## Results

### Demographic and clinical variation in cases of pulmonary NTM

**Fig. 2:**
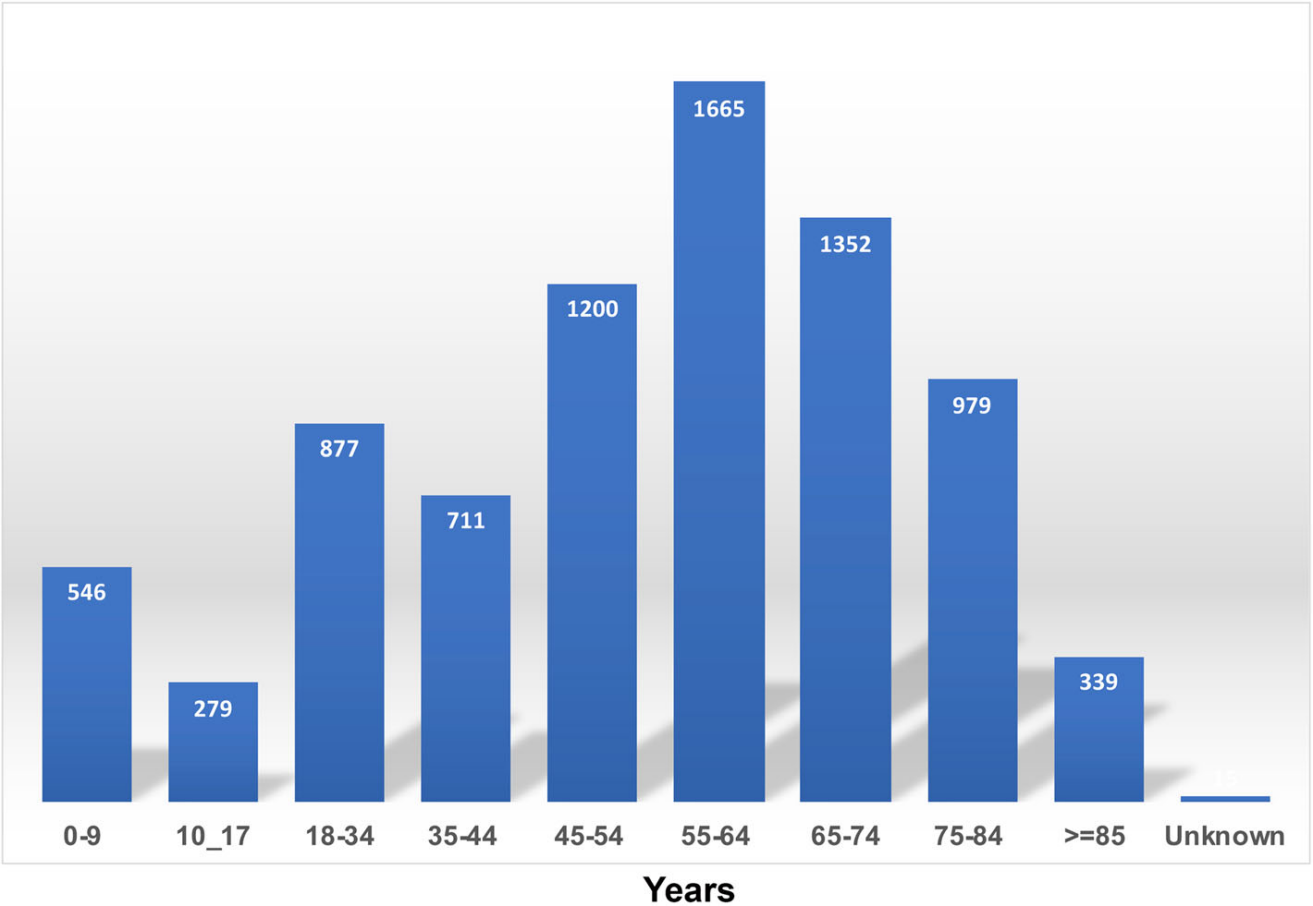
Number of NTM cases per age groups

Of the total pulmonary NTM cases reported during the study period (*N* = 7963), 54% (*N* = 4320) were females. The age at diagnosis showed greatest concentration in age groups greater than 45 years (69.3%). The analysis of incidence and prevalence focused on cases with ages above 45 and above 65 as shown in Fig. 2. The largest racial groups were European American with 58% and African American with 19%. Pulmonary NTM disease was more prevalent in urban residences (91%) compared to rural residences (6%).

Bronchiectasis (31%) and COPD (28%) were the most commonly associated underlying pulmonary diseases among Floridians with NTM disease. Fifty five percent of the cases were immunocompromised, and 44% had neoplastic disorder. The most common NTM-related non-pulmonary comorbidities were gastroesophageal reflux disease (GERD) (42%), and diabetes (25%) as shown in Table 1. The majority of pulmonary NTM cases were untreated, and only 202 (3%) were treated by antibiotics during the study period. Although, it is uncertain if treated was determined with culture negative results. There was no further information in the dataset explaining the reason for no treatment of NTM cases. The highest rate of COPD admissions was recorded in Okeechobee (74.78 per 10,000 admissions). Alachua and Marion counties had relatively low COPD admissions in 2018 (23.65, and 22.05 per 10,000 admissions, respectively) as shown in Supplementary Fig. 1. Alachua and Marion counties had relatively lower age-adjusted rates for COPD emergency room (ER) visits compared to other counties (79.77 and 72.75 in 10,000 ER visits).

**Table 1:** Comorbidity among NTM cases in Florida from 2012 to 2018

Comorbidity	NTM (total number: 7963) (%)
Smoking	500 (6.3%)
Non-smoking	3306 (41.5%)
Immunotherapy	0 (0%)
Immunocompromised	4406 (55.3%)
Steroid therapy > 3 months	74 (0.99%)
Alpha 1 antitrypsin	37 (0.46%)
Bronchiectasis	2445 (30.7%)
Crohn’s Disease	102 (1.3%)
COPD	2207 (27.7%)
Cystic Fibrosis	329 (4.1%)
Diabetes	1980 (24.9%)
GERD	3374 (42.4%)
HIV	1281 (16.1%)
Hyperlipidemia	2993 (37.6%)
Hypertension	3494 (43.9%)
Immunodeficiency	918 (11.5%)
Lung transplant recipient	101 (1.3%)
Neoplasm	3497 (43.9%)
Tuberculosis	841 (10.6%)
Ulcerative colitis	108 (1.4%)

### Prevalence of pulmonary NTM disease

**Fig. 3:**
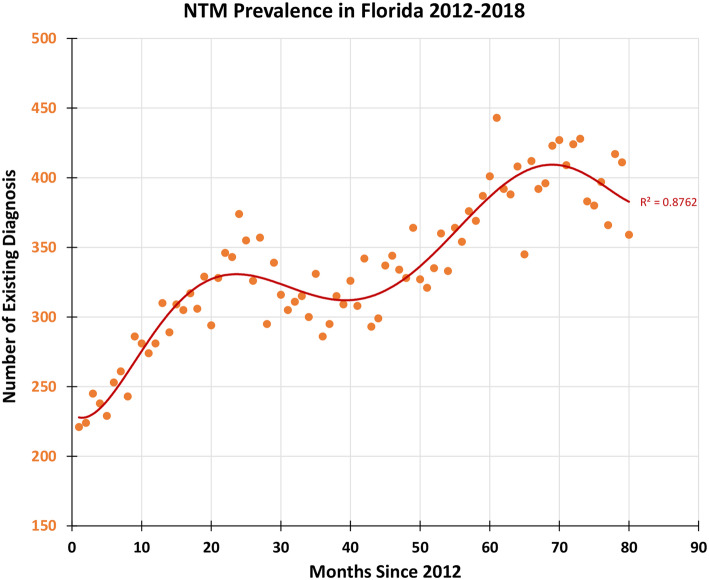
Monthly number of NTM in Florida from 2012 to 2018 (orange circle). The red line is the 6th order polynomial fit with R^2^ = 0.8762

**Fig. 4:**
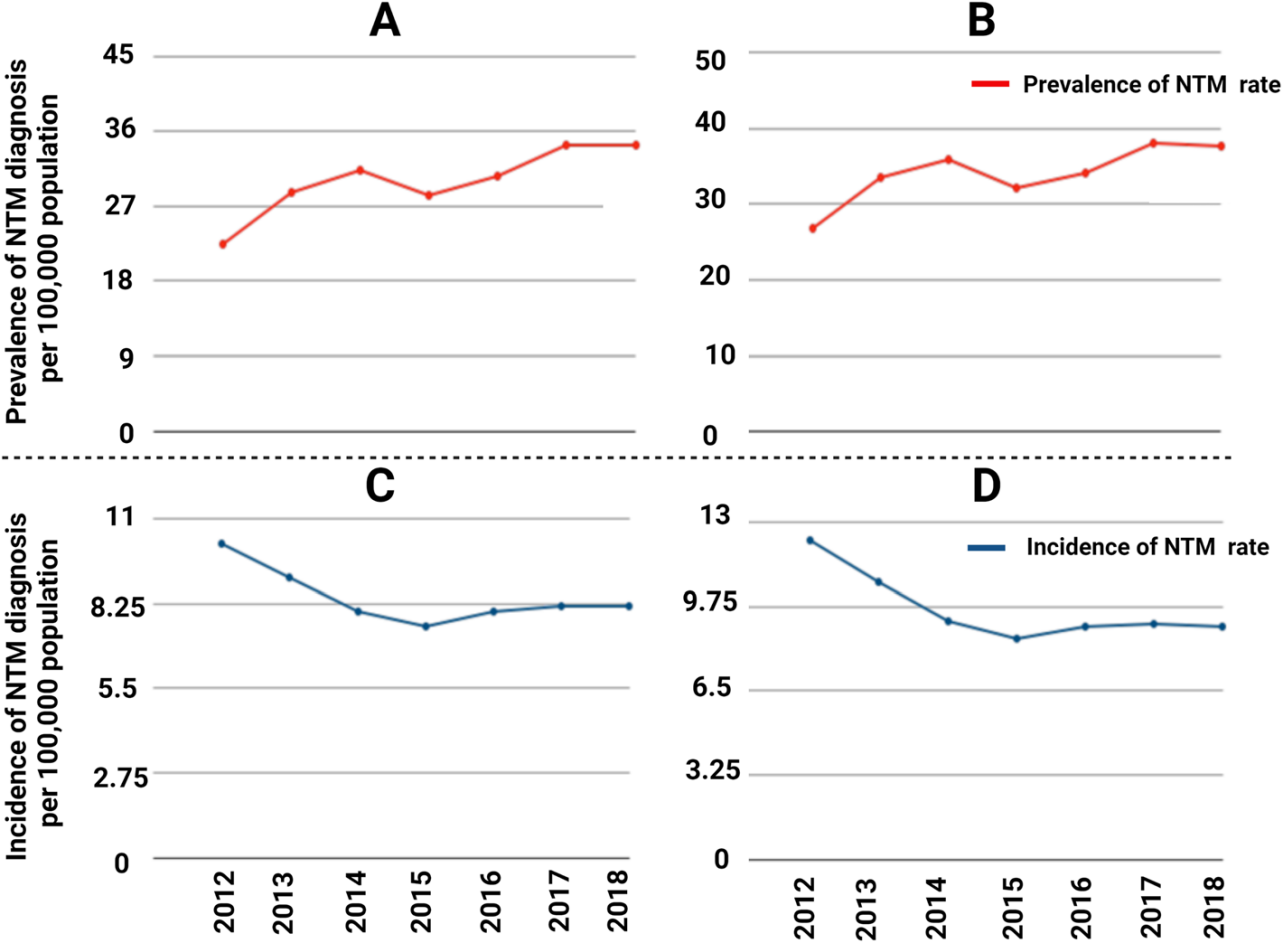
A. Prevalence rate of NTM in age group between 45 to 65 years old, B. Prevalence rate of NTM in age group above 65, C. Incidence rate of NTM in age group between 45 to 65 years old, D. Incidence rate of NTM in age group above 65

In the study period, the prevalence rate for the entire study steadily increased from 14.3 per 100,000 in 2012 to 20.1 per 100,000 in 2015. However, there was an anomalous 8% decrease in the prevalence rate from 2014 to 2015. The prevalence rate rose steadily to 22.6 per 100,000 in 2018. The prevalence rate was adjusted for analysis of ages above 45 and above 65. Similar trends in the prevalence rate were found in the adjusted population. Figure 3 shows an increasing trend in the number of cases of pulmonary NTM in FL (R^2^ = 0.87). The prevalence rate for the age group above 45 rose from 22.5 per 100,000 in 2012 to 34.4 per 100,000 in 2018 (Fig. 4A). There was disruption in the increasing trend with a 9% decrease in the prevalence rate from 2014 to 2015. For the age group greater than 65 years, the prevalence rate rose from 2012 to 2018 from 26.9 per 100,000 to 37.7 per 100,000 (Fig. 4B). There was an 11% decrease in prevalence from 2014 to 2015.

### Incidence of pulmonary NTM disease

The number of new cases of pulmonary NTM in the studied population declined steadily from 6.5 per 100,000 in 2012 to 4.9 per 100,000 in 2015 while from 2015 to 2018, the incidence rate increased slightly to 5.4 per 100,000. The age adjusted incidence rates were analyzed by population age groups above 45 and above 65. For both age groups, the incidence rate experienced the same pattern as found in the entire population. The incidence rate for the age group above 45 decreased from 10.2 per 100,000 in 2012 to 7.5 per 100,000 in 2015 (Fig. 4C). The incidence rate increased to 8 per 100,000 in 2016 and remained fairly constant until 2018. In the age group above 65, the incidence declined from 12.3 per 100,000 in 2012 to 8.5 per 100,000 in 2015 (Fig. 4D). The rate of new cases increased by 6% in 2016 and the incidence rate remained constant around 9 per 100,000 through 2018. The expected trend of increase in NTM along with hurricanes, especially in 2018 following hurricane Irma is not obvious as the major limitation in attributing the cause is delay in diagnosis. Most of the patients presented their infection several days after exposure and seek for medical care was varied among the patients depend on the severity of illness.

### Geographic distribution per period prevalence of pulmonary NTM

**Fig. 5:**
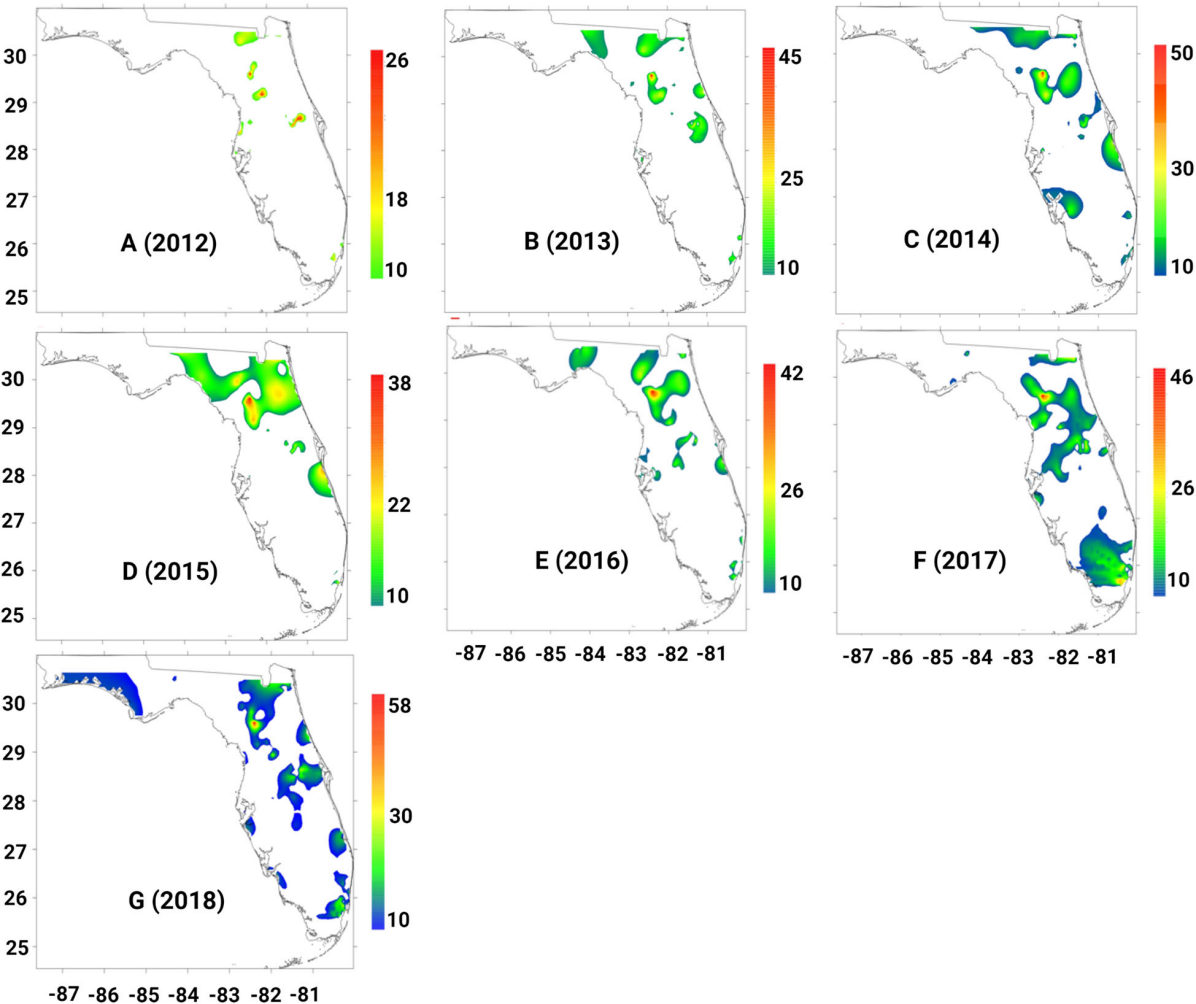
(A-G) Heatmap for the prevalence of NTM cases in Florida by year from 2012 to 2018. Heat maps were generated using Kriging method in R environment (version 4.1.2)

Geographic analysis showed the greatest concentration of cases in the coastal and inland areas of Florida. The zip codes with the highest numbers of pulmonary NTM cases were 32,607,32,608,32,609,34,475, and 34,471. Hotspots were consistently present in Alachua and Marion counties during the study period. Both of the areas of hotspots are inland, not coastal. The yearly heat maps show a gradual expansion of the geographic distribution of pulmonary NTM disease throughout the study period. Of all the maps, those for years 2017 and 2018 (Fig. 5.F and G) show the widest distribution in prevalence. These expansions in distribution parallel the occurrences of two hurricanes: Irma (2017) and Michael (2018). Notably, the 2018 heat map (Fig. 5.G) shows a large area prevalence of NTM in the northwestern, panhandle region of Florida, which had previously been absent in the heat maps of other years.

### Precipitation, surface temperature and hurricane strike in Florida

**Fig. 6:**
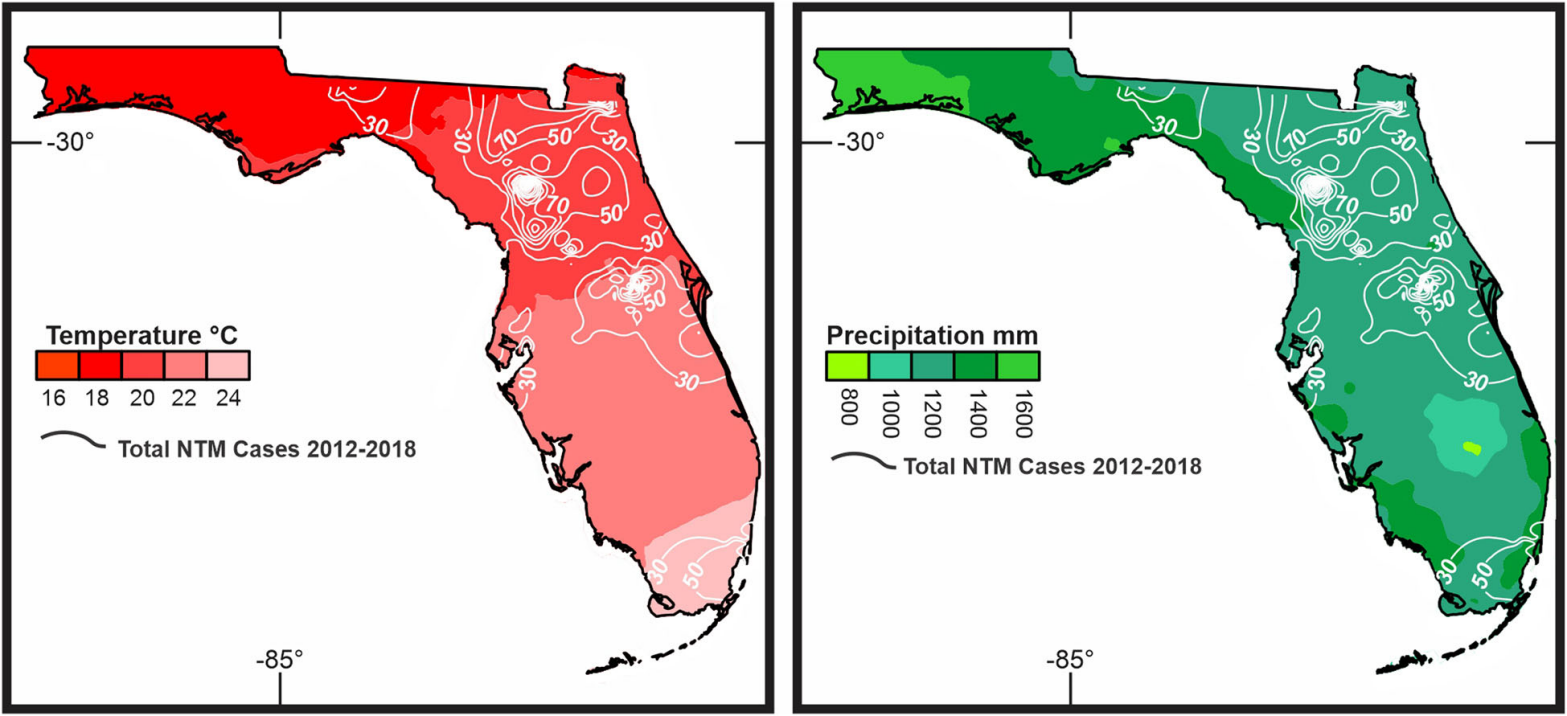
The surface temperature and precipitation maps were overlaid by the distribution of total number of NTM cases in Florida. Precipitation considered as a marker of hurricanes

During the study period, most of the inland counties had lower annual mean precipitation as compared to the panhandle region and coastal areas of FL. Geographically, the mean annual temperature increased gradually from North to South. When compared to the zip codes of NTM cases, a higher prevalence was seen in the region which (Fig. 6) had an annual precipitation rate ranging from 45 to 51 mm and mean annual mean temperature ranging between 18 -21^o^ C.

**Fig. 7:**
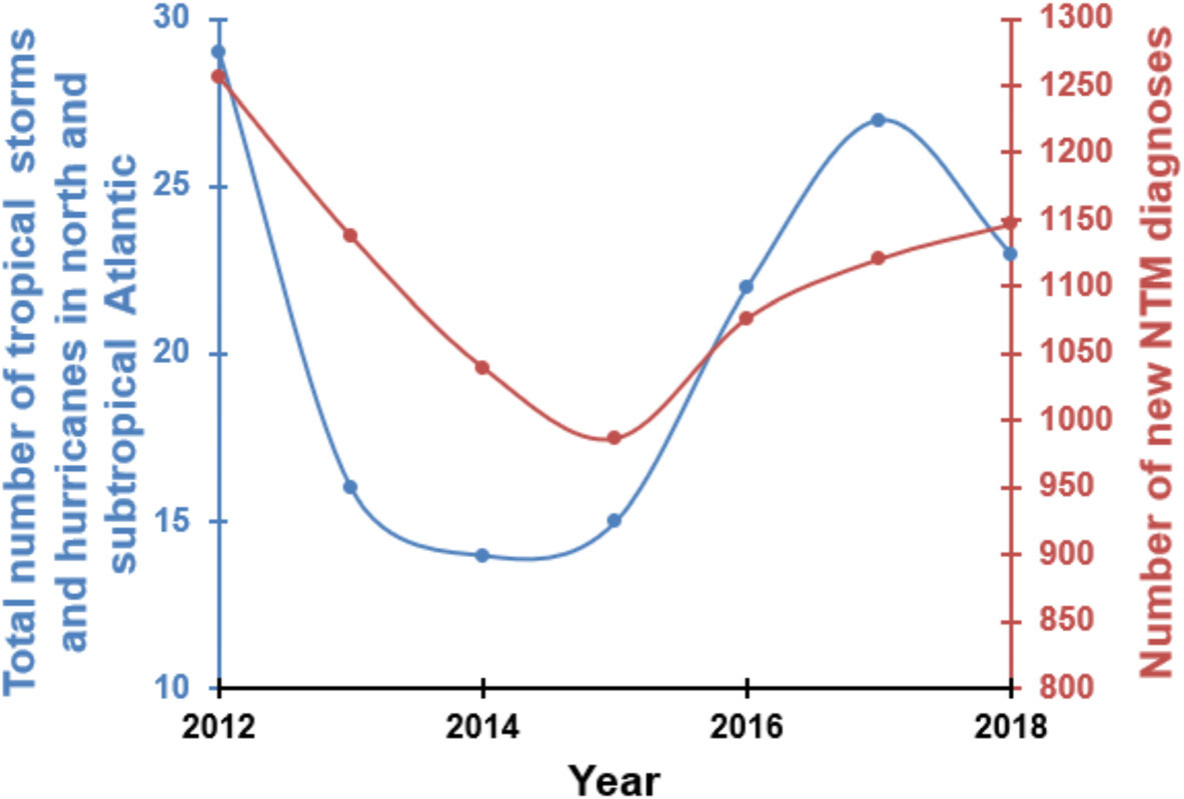
Shows the total number of tropical storms and hurricanes in north and subtropical Atlantic affected Florida state and number of new cases of NTM infections between 2012 and 2018

**Fig. 8:**
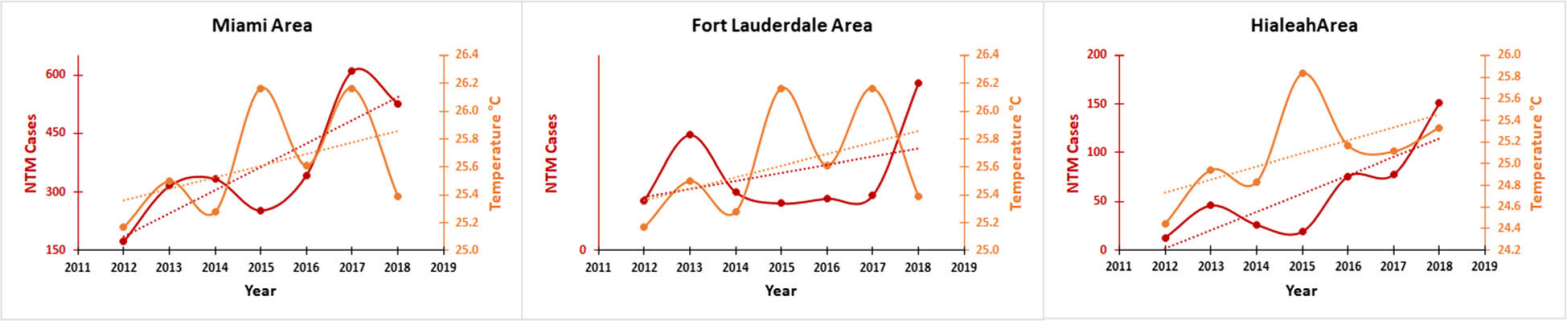
Shows correlation between the increasing number of NTM cases and temperature and NTM cases in Miami through 2012 to 2018

During our study period, 5 major hurricanes made landfall in FL: Hurricanes Isaac (category 1; 2012), Sandy (category 1; 2012), Hermine (category 1; 2016), Irma (category 5; 2017) and Michael (category 5; 2018). However, since Hurricane Isaac passed through the Southern part, affecting only Key West, it might not have affected environment in other area of FL. Figure 7 shows the number of hurricanes and storms affected Florida state between 2012 and 2018. More interestingly, there was an association between the number of hurricanes and storms with the number of new cases of NTM infection (Fig. 7). The highest number of hurricanes and storms was recorded for 2012 including Hurricanes Isaac and Sandy which was associated with the highest new cases of NTM infection in the state. Meanwhile, the lowest number of hurricanes and storms was recorded in 2014 and 2015 which also was associated with the lowest rate of new cases of NTM infection (Fig. 7). In 2016–2018, the number of hurricanes and storms increased notably which also was associated with increasing the number of new cases of NTM infection. Very high prevalence of pulmonary NTM cases was reported in 2017, which was the year after Hurricane Hermine (2016) hit the state. High prevalence of pulmonary NTM cases was seen in 2018, which was the year after Hurricane Irma (2017) hit the state. Hurricane Michael (2018) also occurred late in the study period which may have caused a potential rise in the incidence and prevalence rate of NTM in the years immediately following our study. Figure 8 shows a correlation (R^2^ = 0.14) between the increasing number of NTM cases and temperature and NTM cases in Miami through 2012 to 2018.

## Discussion

The number of tropical storms and hurricanes was very low in 2014 and 2015 but increased in 2016–2018. More interestingly, low number of storms and hurricanes (2014–2015) was associated with the lowest new cases of NTM infection and higher number of storms and hurricanes was associated with higher new cases of NTM infection (2016–2018). The current study found the prevalence rates of pulmonary NTM disease in FL rose during the period between 2012 and 2018, but the prevalence was at lowest level in 2014 and 2015. A higher prevalence of cases was observed in inland areas of the state, which predominantly included Alachua and Marion Counties. However, finding a direct correlation between hurricane strikes and NTM infection rate in some specific locations (i.e., Alachua and Marion Counties) needs another study to monitor hurricanes and NTM infection rate closely for a long period. It is reported that infectious diseases acquired from an extreme weather event or natural disasters are shown to have more complications during the recovery period, especially if individuals are exposed to water-soil mixtures [16].

Thomson et al, found that different environmental variables associated with climate changes such as temperature, rainfall, flooding and drought influence the prevalence of NTM related disease [10]. During and after natural disasters such as hurricanes, storms, earthquakes and tornados, the ecosystem that is normally inhabited by NTM is disrupted by large-scale mixing of ocean water with fresh water and of water with soil, with potentially causing widespread water–soil NTM aerosolization [20]. For example, Honda et al., found a higher number of pulmonary NTM diseases in Louisiana residents following three major hurricanes [16]. We found that the years with more hurricanes (2012, 2016, 2017 and 2018) recorded a higher number of NTM cases (Fig. 7). An association between temperatures and numbers of pulmonary NTM cases was found in the current study. Global mean temperature (GMT) has increased 0.74 °C since pre-industrial times due to effects of climate change and shown a wide range of impacts on ecosystems and species in all regions of the world [21]. Temperature rose from 25 °C in 2012 to 25.4 °C in 2018 (26.2 °C in 2015 and 2017) over our study which also associated with NTM rise (R2 = 0.14-low correlation) (Fig. 6) which potentially reflect the impact of climate change on the rate of NTM; however, further studies with the longer period are needed to measure the real impact of temperature and climate change on NTM disease rate. Our data show that the number of pulmonary NTM cases in FL increased following hurricanes. The current study suggests a possible association between the occurrence of hurricanes in Florida and rise in NTM prevalence and incidence (Fig. 7). During the study period, two hurricanes made landfall on FL, Irma (2017) and Michael (2018); however, their effects may take more time to manifest as higher rates of NTM disease in the data. In addition to the delayed presentation as a possible interference in the data analysis, factors such as increased clinical awareness, changes in diagnostic evaluation, and discrepancies in coding practice may have contributed to the patterns seen in incidence and prevalence of pulmonary NTM. More importantly, more detailed data collection on NTM subspecies could guide further understanding about the increase in certain species following hurricanes, tropical storms or other extreme weather events.

The rise in prevalence parallels the national rise in prevalence rates of pulmonary NTM seen in a recent study [6]. However, given FL’s environmental factors, including extreme weather events, and the high number of elderly residents, FL has the potential to be a super endemic area for NTM in the U.S. In our previous study, we reported that pulmonary NTM disease was more common among postmenopausal women [5, 22]. Notably, in the current study, the distribution of females was mildly more than males with a female to male ratio of 1.19. Park et al. reported a ratio of Korean female to male patients with pulmonary NTM of 1.57 [23]. Similar to prior studies, the highest prevalence of pulmonary NTM was found in the age groups above 45 and 65.

Most common comorbidities associated with pulmonary NTM were bronchiectasis, COPD, diabetes, hypertension, and hyperlipidemia. Nearly half of the cases had a history of cancer or were immunocompromised. These findings are in line with ours and others [24–26]. Interestingly, in the counties with the highest pulmonary NTM cases in FL, the age-adjusted rate of COPD hospitalizations per 10,000 was relatively low compared to other counties. This finding needs further investigation. Only 3% of the patients (202 NTM patients out of 7963 cases) were treated by antibiotics during the study period, however, we could not obtain accurate details for the untreated population. Prolonged period of treatment, non-compliance, failure to report culture negative after treatment, physician’s decision to treat or not treat based on subspecies and symptoms could be some of the possible causes for being untreated.

Earlier we conducted an online survey which included patients diagnosed with pulmonary NTM disease from 46 states in the U. S [27]. We found that the coastal areas were dominant, with higher NTM disease. On the contrary, in FL, the NTM cases were dominant in the inland area. Notably, there were consistent hot spots of pulmonary NTM disease in two inland areas of FL, far from the coasts. These zip codes were clustered in the city of Gainesville and Ocala (Alachua County, and Marion County). A prior study also reported higher pulmonary NTM cases in inland Highland County [7]. Additionally, the transition of the climate from tropical to subtropical around these clusters could be an additional contributing factor [7]. These findings suggest a potential role of environmental factors in the high rate of pulmonary NTM in these two counties and require further investigation in NTM isolation from soil and water, including surface water and drinking water resources.

The major limitation of this study is the validity of the NTM ICD-9 and ICD10 codes. The validity of administrative data on NTM may also vary across Florida. We could not investigate NTM species or laboratory and radiologic data in this study. Therefore, we were unable to validate diagnosis of NTM in the enrolled cases. The treatment rate also was low (3%) and we were not able to validate treatment data too. In addition, ICD codes for NTM include only the more common species including *M. avium complex* and *M. kansasii*. The prevalence of NTM infection was high in 2012 and 2016–2018 (Fig. 7) in Florida while the number of hurricanes strikes also was high too at those years. Our data analysis shows that the increase in the number of hurricane strikes matches the increase in the prevalence of NTM infection in Florida, however, more studies in the long-run are needed to determine if increases in hurricane strikes can result in (causality) increases in NTM infections.

## Conclusion

The higher prevalence of pulmonary NTM in Florida following the hurricanes is likely because of the disruption in the ecosystem. Further investigation is needed to correlate clinical NTM isolates with environmental NTM isolated from air, water, and soil from areas with the highest pulmonary NTM prevalence.

### Highlights

-The prevalence rates of pulmonary NTM disease in FL rose from 2012 to 2018.

-The higher prevalence of pulmonary NTM in FL is possibly associated with hurricanes.

- Pulmonary NTM disease was more prevalent in urban residences (91%) compared to rural residences (6%).

- 97% of pulmonary NTM cases in FL during the study period went untreated.

## Supplementary Information

**Additional file 1: Supplementary Fig. 1**. Shows age-adjusted rate of COPD Hospitalizations per 10,000 (2018). Data acquired from Florida Environmental Public Health Tracking Program. The image was generated by the authors using Microsoft Editor (Office 365).

## Data Availability

The datasets supporting the conclusions of this article are available through direct email to the corresponding author (msm249@med.miami.edu or m.mirsaeidi@ufl.edu).

## Abbreviations

AFB: Acid-fast bacilli
CF: cystic fibrosis
COPD: Chronic obstructive pulmonary disease
ER: Emergency room
FL: Florida
GERD: gastroesophageal reflux disease
GMT: Global mean temperature
LOINC: Logical observation identifiers names and codes
NHC: National Hurricane Center
NOAA: National Oceanic and Atmospheric Administration
NTM: Nontuberculous mycobacteria
TB: tuberculosis
U.S.: United States
