# Supplementary figures and images for: Pulmonary nontuberculous mycobacterial disease in Florida and association with large-scale natural disasters
Source: BMC Public Health. 2021 Nov 10;21:2058. doi: 10.1186/s12889-021-12115-7 (PMC8579656; doi:10.1186/s12889-021-12115-7)

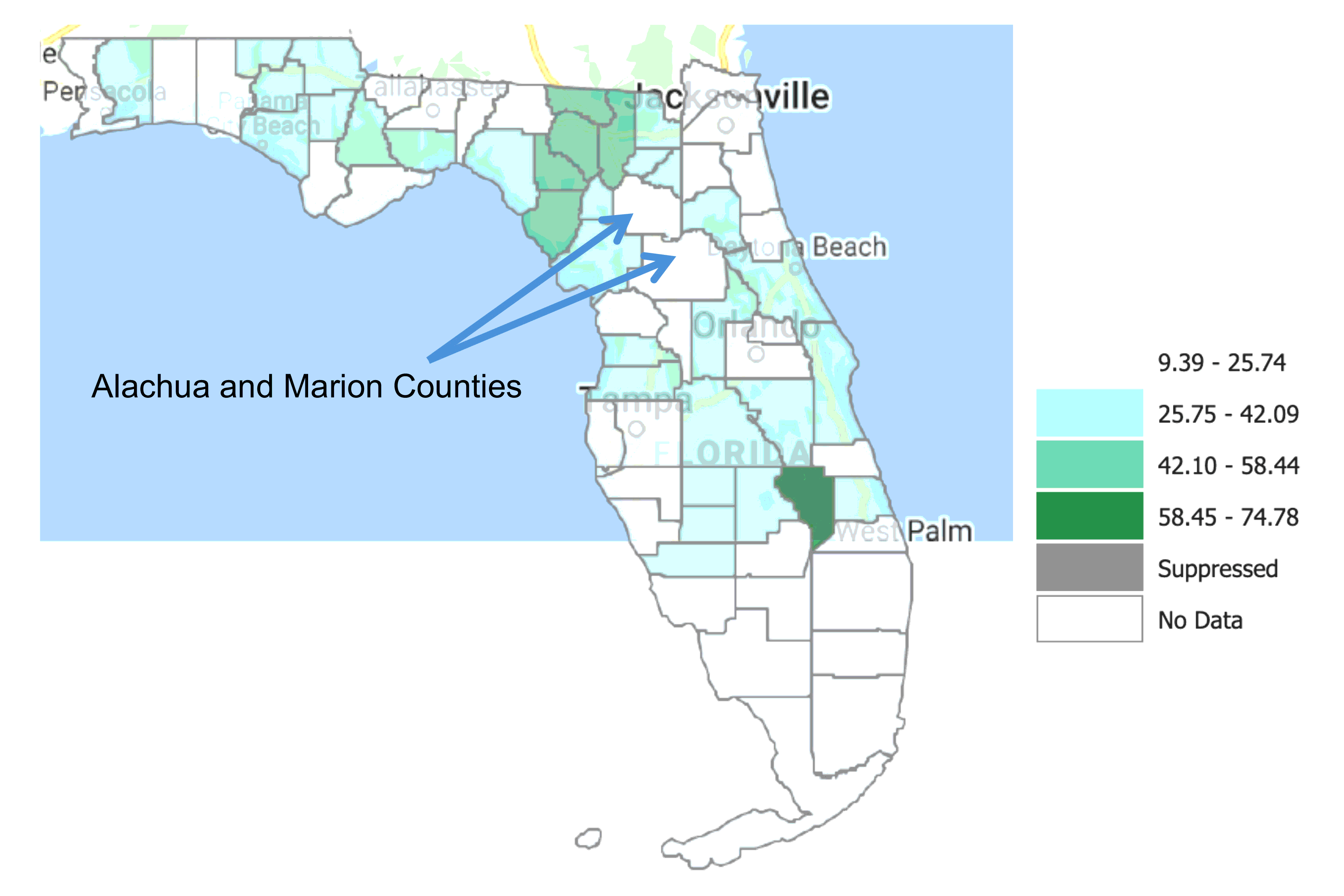

Supplement: Supplementary file 1 — Additional file 1: Supplementary Fig. 1. Shows age-adjusted rate of COPD Hospitalizations per 10,000 (2018). Data acquired from Florida Environmental Public Health Tracking Program. The image was generated by the authors using Microsoft Editor (Office 365). [file 12889_2021_12115_MOESM1_ESM.png]
